# Draft Genome Sequence of *Bacillus marisflavi* TF-11^T^ (JCM 11544), a Carotenoid-Producing Bacterium Isolated from Seawater from a Tidal Flat in the Yellow Sea

**DOI:** 10.1128/genomeA.01451-15

**Published:** 2015-12-10

**Authors:** Jie-ping Wang, Bo Liu, Guo-hong Liu, De-ju Chen, Qian-qian Chen, Yu-jing Zhu, Zheng Chen, Jian-mei Che

**Affiliations:** Agricultural Bio-Resources Research Institute, Fujian Academy of Agricultural Sciences, Fuzhou, Fujian, China

## Abstract

*Bacillus marisflavi* TF-11^T^ (JCM 11544) is a Gram-positive, spore-forming, and carotenoid-producing bacterium isolated from seawater from a tidal flat in the Yellow Sea. Here, we report the first draft genome sequence of *B. marisflavi* TF-11^T^, which comprises 4.31 Mb in 11 scaffolds with a G+C content of 48.57%.

## GENOME ANNOUNCEMENT

The type strain TF-11^T^ of *Bacillus marisflavi*, a Gram-positive or Gram-variable (in older cultures), endospore-forming, and moderately halophilic bacterium, was isolated from seawater from a tidal flat in the Yellow Sea in South Korea (1). So far, the different strains of *B. marisflavi* have been found in a variety of environments, such as rice paddy soils (2), hypersaline microbial mats (3), the solid waste and liquid effluent of an electroplating industry (4), a tropical soil contaminated with petroleum hydrocarbons (5), and different plant rhizospheres (6).

It was uncovered that *B. marisflavi* produces carotenoid with absorption maxima at 455 nm (7), a finding consistent with the pale yellow phenotype of its colonies (1). Some strains of *B. marisflavi*, which inhabit varied plant rhizospheres, displayed the ability to fix nitrogen (2, 6). In environmental biotechnology, *B. marisflavi* was found to be one of the main three members in a lubricant-degrading microbial consortia (5); *B. marisflavi* tolerated chromium concentrations up to 700 mg/liter and performed chromium biosorption quickly and efficiently (4). In biomedical applications, the *Bacillus marisflavi* biomass was used as a reducing and stabilizing agent for the preparation of water-soluble grapheme, which exhibits obvious cytotoxicity against human breast cancer cells (8). Here, we report the draft genome of *B. marisflavi* TF-11^T^, the first released *B. marisflavi* genome sequence.

The genome sequence of *B. marisflavi* TF-11^T^ was obtained by paired-end sequencing on the Illumina Hiseq 2500 system. Two different DNA libraries with insert sizes of 500 and 5,000 bp were constructed and sequenced. After filtering of the 1.21-Gb raw data, the 1.19-Gb clean sequence data were obtained, providing approximately 150-fold coverage. The average DNA G+C content was 48.57%, being in accordance with the previously acquired value of 49 mol% acquired by HPLC determination (1). The reads were assembled via SOAPdenovo software version 2.04 (9). Through the data assembly, 11 scaffolds consisting of 4,312,088 bp were obtained, and the scaffold *N*_50_ was 1,039,146 bp. The average length of the scaffolds was 392,008 bp, and the longest and shortest scaffolds were 1,799,863 bp and 1,254 bp, respectively. Moreover, 92.09% clean reads were aligned back to the genome, by which 99.91% of the genome sequence was covered.

The assembly was uploaded for annotation to the National Center for Biotechnology Information Prokaryotic Genomes Annotation Pipeline version 2.9 (PGAAP) (http://www.ncbi.nlm.nih.gov/genomes/static/Pipeline.html). A total of 4,340 genes were predicted, including 4,143 coding sequences (CDS), 106 pseudogenes, and 84 tRNA and 6 rRNA genes. There were 3,185 and 2,167 genes assigned with COG and KEGG databases, respectively.

### Nucleotide sequence accession numbers.

This whole-genome shotgun project has been deposited at DDBJ/EMBL/GenBank under the accession number LGUE00000000. The version described in this paper is version LGUE01000000.
